# Unmasking Hemiplegic Migraine: A Diagnostic Dilemma in the Shadow of Stroke

**DOI:** 10.7759/cureus.95541

**Published:** 2025-10-27

**Authors:** Kurt C Luchia, Joseph Lajoie

**Affiliations:** 1 Family Medicine, Ross University School of Medicine, Pontiac, USA; 2 Family Medicine, Clarkston Medical Group, Clarkston, USA

**Keywords:** aphasia, cacna1a mutation, cortical spreading depression, hemiplegic migraine, migraine with aura, neuroimaging, prophylactic migraine therapy, sporadic hemiplegic migraine, stroke mimic, unilateral weakness

## Abstract

A rare presentation of a migraine headache is hemiplegic migraine (HM), which can clinically imitate other conditions, including transient ischemic attack (TIA) and cerebrovascular accident (CVA), with unilateral muscle weakness or hemiplegia, making accurate diagnosis challenging. We present a 41-year-old male patient who was admitted with symptoms of a unilateral occipital headache, expressive aphasia, and right-sided motor weakness on two occasions over six days to two different hospitals. CT with and without contrast, echocardiogram, troponin I, EKG, and MRI results were normal. The patient was seen and discharged by neurology to be treated for headaches as an outpatient with butalbital-acetaminophen-caffeine. A diagnosis of sporadic HM was made after a thorough history by the primary care provider and managed conservatively with verapamil. This case highlights the critical role of primary care in synthesizing clinical information and history to establish an accurate diagnosis, underscoring the need for detailed patient evaluation. Raising awareness of HM as part of the differential diagnosis is vital after ruling out life-threatening conditions through imaging and clinical evaluation.

## Introduction

Hemiplegic migraine (HM) is a rare and severe subtype of migraine characterized by temporary paralysis or weakness on one side of the body, known as hemiplegia, during an attack. This condition is classified into two types: familial HM (FHM), which runs in families, and sporadic HM, which occurs in individuals without a family history of the condition [1].

The pathophysiology of HMs involves genetic mutations affecting ion channels and neurotransmitter transport. Known gene variants associated with FHM include CACNA1A, ATP1A2, SCN1A, and PRRT2 [2,3]. These genetic changes lead to cortical spreading depression, a wave of altered neuronal activity that spreads across the brain and disrupts normal nerve cell function [4].

Migraine with aura has also been linked to an elevated risk of ischemic stroke, especially in younger patients and women [5], further complicating the evaluation of HM and underscoring the need to rule out cerebrovascular events before diagnosis.

Clinically, HMs present with symptoms similar to other migraine types but include distinctive features such as muscle weakness, vision changes, numbness, and speech difficulties. These symptoms can last from a few hours to several days and can occasionally be confused with a stroke [1]. This clinical imitation in presentation often delays diagnosis to the patient's detriment [6].

Management of HM is complex and includes both acute and preventive treatments. Acute treatment may involve the use of analgesics and antiemetics, while preventive strategies include the use of anticonvulsants, antidepressants, and calcium channel blockers. The use of triptans in HM is controversial due to concerns about potential vascular side effects, and some experts recommend avoiding them [4].

Overall, HMs require careful diagnosis and management due to their severe and potentially debilitating nature.

## Case presentation

On day one at 3:26 pm, a 41-year-old physically fit male with a history of mixed anxiety and depressive disorder presented to the emergency department of Hospital B. He reported a one-hour history of expressive aphasia and numbness with tingling in his right arm. Two days prior, he experienced a headache that had since resolved. The patient mentioned a similar episode years ago, thought to be a transient ischemic attack (TIA), though no follow-up was conducted, and there had been no recurrences since.

Initial presentation and workup at Hospital B

At presentation, vital signs were hypertensive (142/75 mmHg), tachycardic (111 bpm), afebrile (97.5°F/36.4°C), tachypneic (22 RR), and SpO_2_ 100%. He was alert, oriented, and neurologically intact, with cranial nerves II-XII grossly normal and no focal deficits. His strength was 5/5 in all extremities, and the National Institutes of Health Stroke Scale (NIHSS) score was 0. Initial investigations included a chest X-ray, stroke protocol CT of the head with and without contrast, CBC with differential, comprehensive metabolic panel, drug screen, glucose point-of-care testing, and a 12-lead EKG.

The EKG revealed a regular sinus rhythm at a rate of 87 with no specific ST changes. The CT scan showed no acute findings: gray-white matter differentiation was preserved, ventricles were normal in size, and basal cisterns were patent. The patient's speech improved, but he reported lingering difficulty expressing himself. Sensation in the right arm spontaneously returned after less than three hours. The patient received aspirin and potassium for mild hypokalemia. Imaging of the chest and brain revealed no abnormalities, and the patient was transferred to Hospital CH, where more suitable supportive services are offered with a working diagnosis of TIA or cerebrovascular accident (CVA).

Transfer to Hospital CH

The patient arrived at Hospital CH at 6:54 pm, approximately 3.5 hours after symptom onset. A CT angiogram of the head and neck revealed no stenosis or occlusion in major arteries and no acute perfusion abnormalities. Monitoring and further evaluation continued into the next day. On day two, laboratory results, including troponin I and lipid panels, were within normal limits. Echocardiography demonstrated a normal left ventricular ejection fraction (60%) and no evidence of a patent foramen ovale.

The patient shared that he had a high-stress job with rapid deadlines and recalled a similar episode in his late 20s that resolved spontaneously. He expressed relief at having no deficits and a desire to return home.

MRI and discharge from Hospital CH

**Figure 1:**
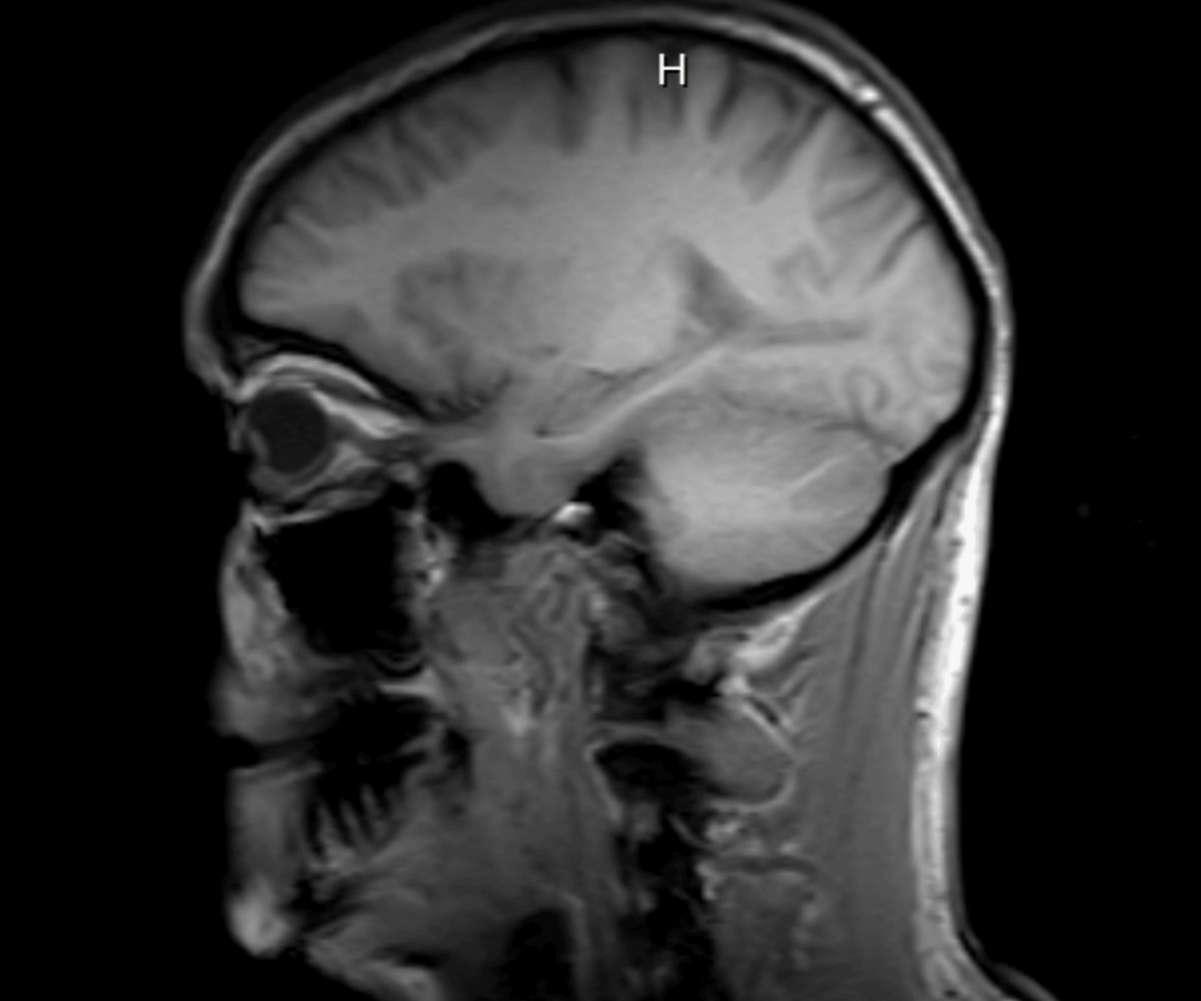
MRI sagittal view T1-weighted. No acute intracranial process.

**Figure 2:**
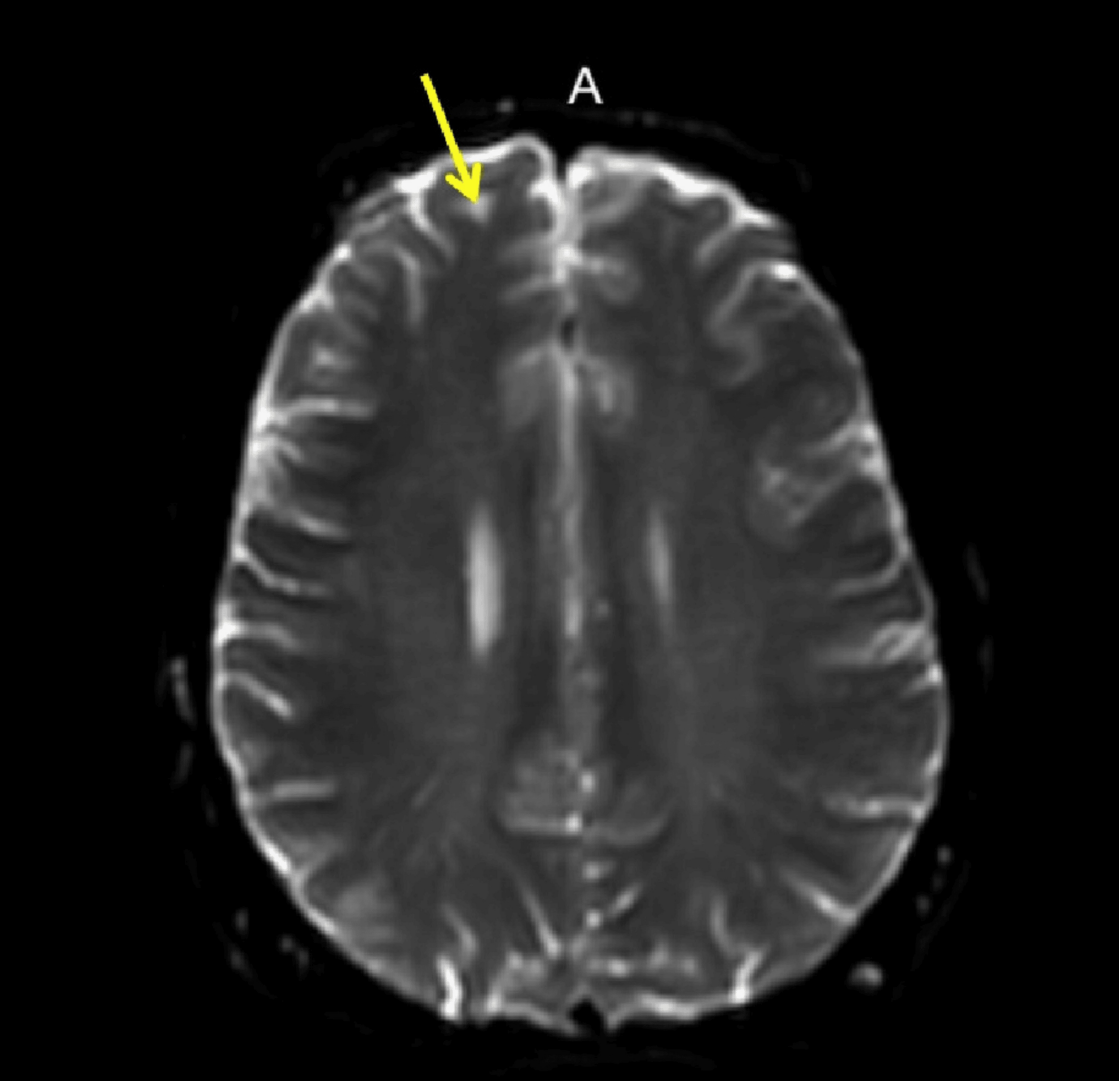
MRI axial view T2-weighted. There is a sub-centimeter focus of the hyperintense signal (arrow) within the white matter of the right frontal lobe (nonspecific) and could be sequela of migraine headaches or related to chronic small vessel ischemic change.

**Figure 3:**
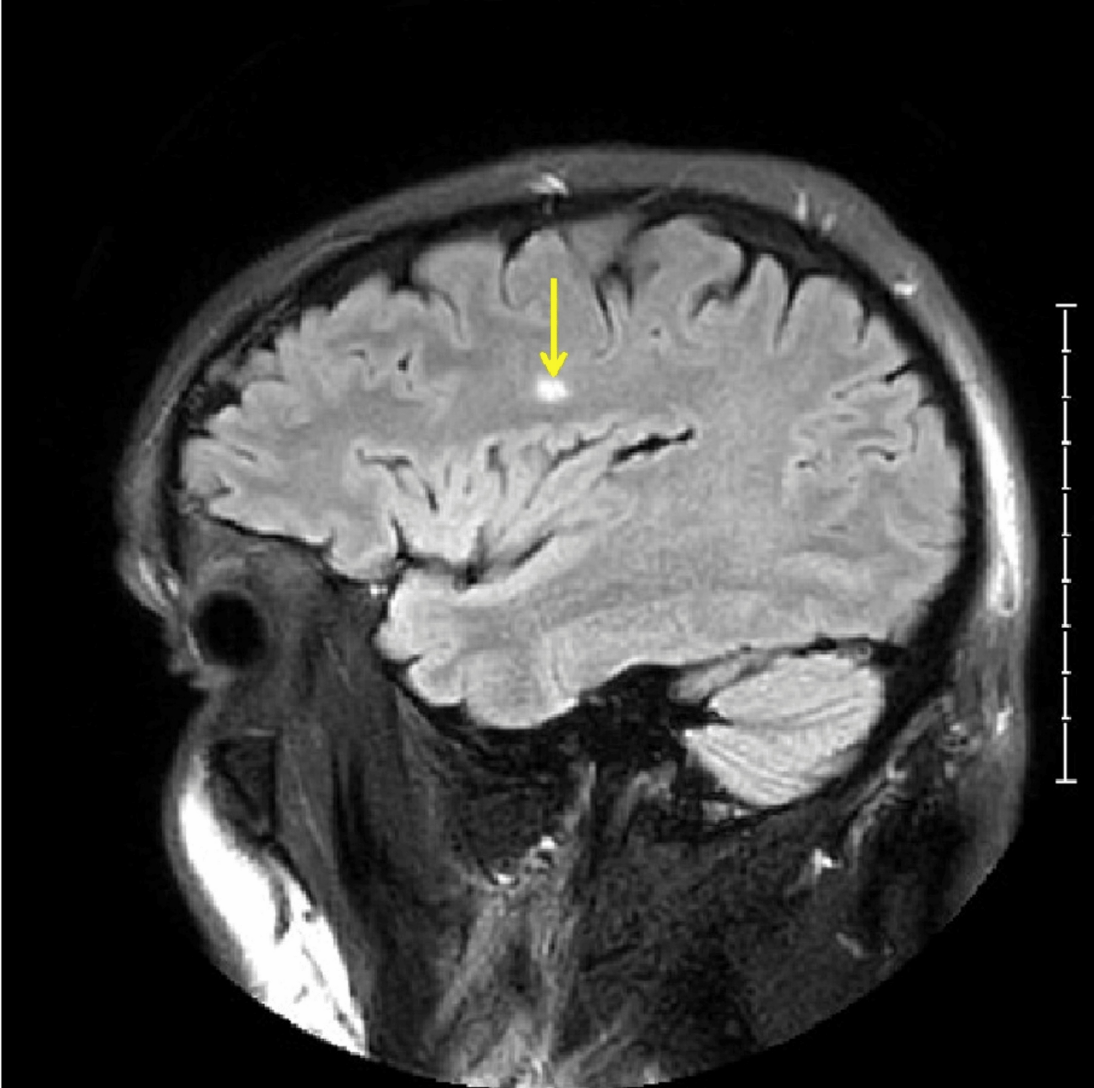
Brain MRI demonstrating a small focus of non-specific white matter hyperintensity in a patient with hemiplegic migraine. Sagittal FLAIR sequence showing a 7 mm hyperintense lesion within the deep white matter of the right frontal lobe (arrow). There is no associated diffusion restriction or mass effect. Such non-specific findings may represent sequelae of migraine-related vasogenic changes or chronic small-vessel ischemic alterations (Kellam score: 14.) FLAIR: Fluid-Attenuated Inversion Recovery

An MRI performed on day three showed no acute infarction, intracranial hemorrhage, or mass lesion (Figure 1). T2-weighted sequences demonstrated nonspecific punctate foci of white matter signal alteration in the frontal and parietal lobes, attributed to chronic ischemic changes (Figure 2). On fluid-attenuated inversion recovery (FLAIR) imaging, a small 7 mm hyperintense focus was noted within the deep white matter of the right frontal lobe, without diffusion restriction or mass effect (Figure 3). FLAIR imaging, which nulls cerebrospinal fluid signal to better delineate parenchymal abnormalities, is particularly sensitive to subtle cortical and subcortical lesions associated with migraine-related vasogenic edema or chronic small-vessel ischemic change. With no further events or significant findings, the patient was discharged on day three with a diagnosis of stroke-like symptoms, continuing aspirin and statin therapy.

Presentation at Hospital MC

On day four, at 10:59 am, the patient presented to Hospital MC with slurred speech and right-hand numbness that began 40 minutes prior. He had been discharged the day before and had been compliant with aspirin and statin therapy. On examination, he was oriented, with no focal deficits, negative Babinski signs, and normal coagulation studies. A brain CT and EKG were unremarkable. Stroke, subarachnoid hemorrhage, and brain aneurysm were considered but deemed unlikely due to negative imaging. The patient was transferred to Hospital MO for an available inpatient neurological support and assessment.

Evaluation at Hospital MO

At Hospital MO, the patient reported vision changes described as "squiggly lines," a circular aura, a bilateral headache, and numbness in his hands, throat, and chest. Neurology noted that his deficits did not align with specific arterial territories, making CVA less likely. The patient’s anxiety and recent outpatient psychiatric evaluation were noted. Another MRI confirmed no acute findings but showed minor changes consistent with headaches.

Neurology diagnosed the patient with stroke-like symptoms and prescribed outpatient headache management. He was discharged on day six with aspirin, a statin, and medications for headache relief, including butalbital-acetaminophen-caffeine. Additional prescriptions included mirtazapine and venlafaxine.

Follow-up and diagnosis

Two weeks after discharge, the patient saw his primary care physician and reported no further episodes. He described his initial symptoms, which began with a lower right visual field defect and progressed to bilateral arm numbness, expressive aphasia, and headache. A detailed history revealed similar episodes in his late 20s, including numbness and transient visual defects.

With review of the previously completed extensive imaging and laboratory, as well as a complete history taken in the office at the outpatient follow-up appointment, the diagnosis of HM was made. The criteria for this diagnosis include at least two attacks featuring reversible aura symptoms, such as visual, motor, speech, or language deficits. Minor criteria - such as gradual symptom spread, succession of symptoms, unilateral aura, and symptom duration of 5-60 minutes - were also satisfied.

The patient was prescribed 120 mg of extended-release verapamil daily to manage HMs. This diagnosis explained his recurring episodes, including their transient nature and associated neurological symptoms.

## Discussion

HM often presents with clinical signs that mimic those of strokes or TIAs [6]. Once a thorough workup, including standard protocols and imaging, rules out a stroke, a diagnosis of HM should be considered. A comprehensive history that identifies transient visual, motor, and sensory deficits characteristic of this rare migraine variant is essential for proper diagnosis and treatment [7]. According to the International Classification of Headache Disorders 3rd Edition (ICHD-3), diagnosis requires ≥2 attacks with reversible motor aura plus at least one additional aura symptom [8]. This patient met the criteria with recurrent episodes of motor weakness, aphasia, and visual aura. The patient’s expressive aphasia suggests transient cortical spreading depression affecting Broca’s area and adjacent premotor regions. The right-sided weakness likely reflects involvement of upper motor neuron pathways in the left hemisphere. As the brain parenchyma itself is pain-insensitive, headache symptoms arise from activation of trigeminovascular pathways, with meningeal blood vessel irritation being the probable source. Ion channel dysfunction leads to cortical spreading depression, which activates trigeminal afferents. These afferents innervate meningeal blood vessels, leading to the release of vasoactive peptides and headache generation [4,9].

Due to its rarity, HM is frequently overlooked, and prevalence data in the United States are scarce. A Danish study estimates the prevalence of HM at approximately one in 10,000, with a male-to-female ratio of 1:3 [10]. This epidemiological context makes severe cases in males, like the present case, infrequent. Rapid onset of HM can mimic a stroke, with bilateral involvement seen in about 35% of cases [11]. A study among United States military personnel found no incidence from 1997 to 2007 and 597 cases from 2008 to 2016, suggesting a higher prevalence and incidence in post-9/11 service members, warranting further research into potential causes [12].

In addition to stroke and TIA, cerebral amyloid angiopathy (CAA)-related transient focal neurological episodes, often termed “amyloid spells,” should also be considered in the differential. These events may present with transient aphasia, hemiparesis, or sensory deficits that closely resemble HM [13]. While CAA is more commonly described in older adults, similar presentations have been reported in middle-aged patients, particularly when MRI reveals chronic white matter changes. However, this patient’s history of a nearly identical episode in his 20s, decades before the current presentation, argues against CAA as a unifying diagnosis. The absence of microhemorrhages on MRI further reduces the likelihood, although consideration of this finding underscores the importance of comprehensive neuroimaging and clinical correlation [13]. Differential diagnoses also include other channelopathies, such as episodic ataxia and familial epilepsy syndromes [2,3]. A focal seizure with postictal weakness could not be fully excluded, as no EEG was obtained; however, the patient had no prior history of seizures, and no postictal confusion or prolonged recovery was witnessed during the episode. EEG is not part of the standard acute stroke workup, which further supports the clinical decision-making in this case [5].

Stress likely contributed but should be considered alongside other common precipitants, such as sleep disruption, hormonal changes, and environmental triggers. In this patient, high occupational stress may have acted as one of several contributing factors [14]. The patient's first episode in his late 20s occurred during a vacation abroad, with the first recurrence on a weekend, consistent with this pattern. His age of onset aligns with the typical presentation at 37.8 ± 19.1 years [11]. HM generally becomes less severe and presents as typical migraines after age 50 [1]. The patient's history of moderate anxiety and moderately severe depression, as indicated by recent Generalized Anxiety Disorder (GAD-7) and Patient Health Questionnaire (PHQ-9) scores, suggests that stress was a likely contributor among multiple potential precipitating factors to his clinical presentation. Continued treatment and stress management will be crucial in preventing future episodes.

Treatment for HM includes managing acute attacks and preventing future ones. Nonsteroidal anti-inflammatory drugs (NSAIDs) and antiemetics are commonly used during acute episodes, while intranasal ketamine can be effective for familial HM [9]. Preventive measures often involve medications such as verapamil, flunarizine, and acetazolamide, with lamotrigine being an option for those with more pronounced aura symptoms [1,9]. It is generally advised to avoid triptans and ergotamines due to their potential risk of causing cerebral vasoconstriction [9]. Additionally, identifying and avoiding personal triggers is essential for effective management.

Familial HM has an autosomal dominant inheritance pattern with a penetrance as low as 63% [9]. Presentations can vary widely, from migraines with aura involving sensory, aphasic, or visual disturbances to coma and confusion, with or without permanent cerebellar symptoms such as ataxia, nystagmus, or dysarthria [11]. Given this variability, familial connections can be challenging to determine. In this case, the patient's family history was insignificant for signs of HM or cerebellar involvement, although his brother reported migraines with aura. This lack of a possible inheritance suggests a sporadic HM, highlighting the importance of a thorough patient history for accurate diagnosis. This case was classified as sporadic HM because the patient had no family history of motor aura or hemiplegia. Epidemiological data indicate sporadic HM accounts for approximately half of all HM cases [10]. The patient did have a sibling with a history of non-HM with aura, but no cluster of symptoms of HM. Because first- and second-degree relatives shared no common cluster of symptoms, gene testing was not warranted.

While HM was the favored diagnosis, some diagnostic uncertainty remains due to a lack of EEG and limited imaging. Stroke, focal seizures, and CAA remain important considerations in similar presentations. By ruling out stroke and TIA, and then considering HM, clinicians can avoid misdiagnosis and ensure appropriate treatment. Raising awareness about HM and emphasizing the importance of detailed patient histories are crucial steps in improving outcomes for individuals affected by this rare condition.

## Conclusions

HMs present a significant diagnostic challenge due to their clinical similarity to acute neurologic events, such as stroke and TIA. Timely exclusion of these life-threatening conditions through appropriate imaging and clinical assessment is essential. Once ruled out, HM should be considered, particularly in patients with a personal or family history of migraines. Awareness of hallmark features - including transient hemiplegia, aphasia, and other neurological deficits - is critical for accurate diagnosis. A detailed patient history remains a cornerstone in distinguishing HM from other serious conditions. Improved recognition and understanding of this rare migraine subtype can lead to more appropriate management strategies, patient education, and, ultimately, better clinical outcomes.
